# Editorial: Cardiovascular risks in cardiovascular-kidney-metabolic syndrome: mechanisms and therapies

**DOI:** 10.3389/fendo.2026.1861380

**Published:** 2026-05-01

**Authors:** Ruoyu Zhou, Bo Zhang, Weihao Wang, Jiyuan Piao, Wanlu Ma

**Affiliations:** 1 Department of Endocrinology, China-Japan Friendship Hospital, Beijing, China; 2 Department of Endocrinology, Beijing Hospital, Beijing, China; 3 Faculty of Medicine & Dentistry-Medicine Department, University of Alberta, Edmonton, AB, Canada

**Keywords:** CKM syndrome, inflammation, management, mechanism, therapy

## Introduction

In 2023, the American Heart Association (AHA) introduced a new way of how three major health problems — metabolic disorders, kidney disease, and heart disease — are deeply connected. This framework is called cardiovascular-kidney-metabolic (CKM) syndrome. Rather than treating these conditions separately, doctors and researchers now recognize that they work together to make each other worse (1, 2). CKM syndrome covers a wide range of conditions, including obesity, type 2 diabetes, chronic kidney disease, heart failure, coronary artery disease, stroke, atrial fibrillation, and peripheral artery disease (1, 3). When these conditions occur together, the risk of serious heart and kidney problems becomes much greater than if any one of them existed alone (2). These conditions do not simply coexist — they actively fuel one another. The body becomes insulin resistance, chronic inflammation, endothelial dysfunction, and neurohormonal dysregulation. Together, these processes create a harmful cycle that is difficult to break. CKM syndrome most commonly originates from excess and dysfunctional adipose tissue, particularly visceral fat, which secretes proinflammatory and prooxidative mediators that damage arterial, myocardial, and renal tissues, reduce insulin sensitivity (4, 5). When the liver also becomes affected by fat buildup — a condition now called metabolic dysfunction-associated steatotic liver disease — the inflammation and damage become even worse (3, 6). All of these processes ultimately share the same damaging effects on the body: hemodynamic dysregulation, atherogenesis, thrombosis, and progressive cardiac and renal fibrosis. Over time, these changes quietly accumulate and set the stage for serious heart disease — even before any obvious symptoms appear. This Research Topic assembles 16 original research articles, reviews, and clinical studies that collectively advance our understanding of cardiovascular risk within the CKM spectrum, spanning novel biomarkers, pathophysiological mechanisms, and therapeutic interventions.

## Novel biomarkers and risk stratification

A major theme across this Research Topic is the identification and validation of composite metabolic-inflammatory biomarkers that outperform traditional risk factors in predicting cardiovascular and renal outcomes. The triglyceride-glucose (TyG) index and its derivatives feature prominently. Zhang demonstrated that the high-sensitivity C-reactive protein-triglyceride glucose index (CTI), a novel composite marker integrating inflammation and insulin resistance, is independently associated with CKD prevalence, reinforcing the concept that metabolic inflammation is a central driver of kidney damage (Zhang et al.). In a complementary analysis, one study examined the TyG-waist-to-height ratio and its association with CVD in Chinese adults with sarcopenia, highlighting that body composition modifies the metabolic-cardiovascular relationship (Huang et al.). The TyG index was further shown to predict mortality in congestive heart failure patients with diabetes (Yu et al.), and to exhibit a U-shaped association with acute kidney injury in critically ill children with cardiac diseases (Du et al.), underscoring the complexity and non-linearity of metabolic risk across different populations and clinical settings.

Beyond TyG-based indices, several contributions explored alternative biomarkers. One study investigated the albumin-corrected anion gap (ACAG) as a predictor of hyperuricemia and its mediating role in cardiovascular risk through triglyceride and non-HDL cholesterol pathways (Yan et al.). Another examined the pan-immune-inflammation value and its cross-sectional and longitudinal associations with serum uric acid (Lu et al.), providing evidence that systemic immune-inflammatory status contributes to urate dysregulation, a recognized contributor to CKM progression. The joint impact of serum urate, renal function, and genetic susceptibility on coronary heart disease and ischemic stroke risk was explored in a population-based study (Guo et al.), revealing important gene-environment interactions that modulate cardiovascular vulnerability within the CKM framework. Furthermore, the relationship between cholesterol metabolism and insulin sensitivity was examined in young adults (Sigudu et al.), providing early-life evidence of the metabolic-cardiovascular nexus.

## Mechanistic insights: from inflammation to organ cross-talk

The pathophysiological underpinnings of CKM syndrome were addressed from multiple angles. One study identified monocyte human leukocyte antigen-DR (HLA-DR) as a mediator of diabetic nephropathy progression (Wang et al.), offering a promising immunological therapeutic target at the intersection of metabolic and renal disease. The integrative analysis of cardiac autonomic neuropathy and nephropathy risk using SUDOSCAN in type 2 diabetes patients (Cobuz et al.) elegantly demonstrated the concurrent involvement of cardiac and renal autonomic systems, supporting the concept of multi-organ neurological cross-talk in CKM syndrome. The study on left atrial systolic function improvement after surgical reduction of high-flow arteriovenous fistula (Lejsek et al.) provided direct clinical evidence of the hemodynamic burden imposed by vascular access on cardiac structure in CKD patients, illustrating the bidirectional cardiovascular-kidney interaction that is central to the CKM construct.

## Therapeutic strategies: from pharmacotherapy to integrated management

Several contributions addressed therapeutic interventions with relevance across multiple CKM components. A comprehensive review on mechanism-guided pharmacotherapy for cardiometabolic multimorbidity (Dong et al.) provided a framework for phenotype-prioritized treatment, advocating for individualized therapeutic algorithms that match drug mechanisms to dominant pathophysiological drivers. One study revealed the impact of SGLT2 inhibitors on uric acid levels in a retrospective multicenter cohort (Habeeb et al.), adding to the growing evidence that SGLT2 inhibitors exert pleiotropic benefits extending beyond glycemic control to urate metabolism and potentially to cardiovascular and renal protection. The comparative effectiveness of pemafibrate versus bezafibrate on hepatic and vascular endothelial function in patients with coronary artery disease and metabolic dysfunction-associated steatotic liver disease (Nakamura et al.) addressed the emerging recognition that hepatic steatosis is not merely a metabolic comorbidity but an active participant in cardiovascular risk amplification within CKM syndrome.

In patients with end-stage renal disease, a randomized controlled trial evaluated niacin’s effects on lipoprotein(a) and biochemical markers in hemodialysis patients (Galal et al.), targeting a notoriously treatment-resistant cardiovascular risk factor in a population with extreme CKM burden. The finding that antidiabetic and lipid-lowering therapies modify the association between the TyG index and acute kidney injury in critically ill patients with coronary artery disease (Yang et al.) provided important evidence that pharmacological intervention can attenuate the metabolic-renal-cardiovascular cascade even in acute clinical settings.

## Conclusions and future directions

The studies assembled in this Research Topic collectively reinforce the conceptual validity of the CKM syndrome framework and demonstrate its translational relevance. Several cross-cutting insights emerge. First, composite biomarkers integrating metabolic, inflammatory, and renal parameters consistently outperform single markers in risk prediction, supporting the move toward multi-dimensional risk assessment in clinical practice. Second, the pathophysiology of CKM syndrome is mediated by complex organ cross-talk involving the kidney, heart, liver, immune system, and autonomic nervous system, necessitating interdisciplinary research approaches. Third, existing pharmacotherapies—particularly SGLT2 inhibitors, fibrate derivatives, and lipid-lowering agents—demonstrate benefits that span multiple CKM components, supporting the use of drugs with pleiotropic effects rather than organ-specific monotherapy.

Important gaps remain. Most studies in this Research Topic are cross-sectional or retrospective, limiting causal inference. Prospective, multicenter studies with standardized CKM staging and longitudinal follow-up are needed to validate the biomarkers and therapeutic strategies identified here. The development of integrated care models that break down traditional disciplinary silos between endocrinology, nephrology, and cardiology will be essential for translating the CKM concept into improved patient outcomes. We hope that the contributions to this Research Topic will stimulate further investigation and ultimately inform evidence-based, patient-centered management of this complex and consequential syndrome.

## References

[1] Ndumele CE Rangaswami J Chow SL Neeland IJ Tuttle KR Khan SS . Cardiovascular-kidney-metabolic health: A presidential advisory from the American Heart Association. Circulation. (2023) 148:1606–35. doi: 10.1161/cir.0000000000001184. PMID: 37807924

[2] Khan SS Coresh J Pencina MJ Ndumele CE Rangaswami J Chow SL . Novel prediction equations for absolute risk assessment of total cardiovascular disease incorporating cardiovascular-kidney-metabolic health: A scientific statement from the American Heart Association. Circulation. (2023) 148:1982–2004. doi: 10.1161/cir.0000000000001191. PMID: 37947094

[3] Ndumele CE Neeland IJ Tuttle KR Chow SL Mathew RO Khan SS . A synopsis of the evidence for the science and clinical management of cardiovascular-kidney-metabolic (CKM) syndrome: A scientific statement from the American Heart Association. Circulation. (2023) 148:1636–64. doi: 10.1161/cir.0000000000001186. PMID: 37807920

[4] Sebastian SA Padda I Johal G . Cardiovascular-kidney-metabolic (CKM) syndrome: A state-of-the-art review. Curr Probl Cardiol. (2024) 49:102344. doi: 10.1016/j.cpcardiol.2023.102344. PMID: 38103820

[5] Jang KW Hur J Lee DW Kim SR . Metabolic syndrome, kidney-related adiposity, and kidney microcirculation: Unraveling the damage. Biomedicines. (2024) 12:2706. doi: 10.3390/biomedicines12122706. PMID: 39767613 PMC11673429

[6] Sandireddy R Sakthivel S Gupta P Behari J Tripathi M Singh BK . Systemic impacts of metabolic dysfunction-associated steatotic liver disease (MASLD) and metabolic dysfunction-associated steatohepatitis (MASH) on heart, muscle, and kidney related diseases. Front Cell Dev Biol. (2024) 12:1433857. doi: 10.3389/fcell.2024.1433857. PMID: 39086662 PMC11289778

